# The Effectiveness of *Scutellaria baicalensis* on Migraine: Implications from Clinical Use and Experimental Proof

**DOI:** 10.1155/2021/8707280

**Published:** 2021-01-06

**Authors:** Chung-Chih Liao, Ke-Ru Liao, Cheng-Li Lin, Jung-Miao Li

**Affiliations:** ^1^Graduate Institute of Chinese Medicine, College of Chinese Medicine, China Medical University, Taichung 40402, Taiwan; ^2^Department of Neurology, Yuanlin Christian Hospital, Yuanlin 51052, Taiwan; ^3^Management Office for Health Data, China Medical University Hospital, Taichung 40447, Taiwan; ^4^College of Medicine, China Medical University, Taichung 40402, Taiwan; ^5^Department of Chinese Medicine, Show Chwan Memorial Hospital, Changhua 50008, Taiwan

## Abstract

**Background:**

*Scutellaria baicalensis* (SB), a traditional Chinese medicine, is commonly used for the treatment of inflammatory and painful conditions. The purpose of the present study was to examine the effects of SB on migraine.

**Materials and Methods:**

We examined the clinical applications of SB based on the data obtained from Taiwan's National Health Insurance Research Database and confirmed that it was frequently used in Taiwan for the treatment of headaches. An experimental migraine model was established in rats by an intraperitoneal injection of nitroglycerin (NTG, 10 mg/kg). Pretreatment with SB was given orally 30 min before NTG administration. The rats were subjected to migraine-related behaviour tests that were video-recorded and analysed using EthoVision XT 12.0 software.

**Results:**

The frequency of exploratory and locomotor behaviour was comparatively lower in the NTG group than that in the control group, while the frequency of resting and grooming behaviour increased. These phenomena were ameliorated by pretreatment with 1.0 g/kg SB. The total time spent on the smooth surface was longer in the NTG group than that in the control group, but the time was shortened by pretreatment with 1.0 g/kg SB.

**Conclusions:**

Pretreatment with 1.0 g/kg SB relieved migraine-related behaviours in the experimental NTG-induced migraine model. The outcome therefore demonstrated that pretreatment with 1.0 g/kg SB is beneficial for migraine treatment.

## 1. Introduction

Migraine is a prevalent and complex neurological disorder, characterized by recurrent unilateral, pulsating, moderate-to-severe pain, aggravated by routine physical activity, and associated with nausea, photophobia, or phonophobia [1]. According to large-scale recent research, migraine affects approximately 12% of the population worldwide and has a higher prevalence among the female population, school/college students, and urban residents [2, 3].

Traditional Chinese medicine (TCM) has been used for the treatment of migraine for thousands of years by using TCM theory experience. Recently, a large body of basic and clinical research confirmed the scientific benefit of TCM for the treatment of migraine [4–7]. The root of *Scutellaria baicalensis* (SB, known in Chinese medicine as Huang Qin) is a TCM herb, distributed in countries such as China, Japan, North Korea, Russia, and Mongolia [8]. It is known, from the experience of traditional Chinese doctors, to have analgesic, anti-inflammatory, and neuroprotective effects [9, 10]. Migraine is now known as an inflammatory neurovascular disorder [11]. Although SB has anti-inflammatory and neuroprotective potency, clinical or experimental research on SB in TCM academia for the treatment of migraine has hardly been reported.

As a result, the present study first explored the clinical use of SB in Taiwan. Systemic administration of nitroglycerin (NTG) in rodents to induce hyperalgesia or migraine-related behaviours is one of the most widely accepted experimental migraine models [12–15]. Thereafter, we studied the effects of SB on migraine by behavioural analysis of an NTG-induced migraine rat model.

## 2. Materials and Methods

### 2.1. Clinical Applications of SB

The National Health Insurance program in Taiwan, which is an integration of all public insurance systems, offers insurance for conventional Western medicine and TCM [16, 17]. Detailed information about TCM utilization was collected from the National Health Insurance Research Database (NHIRD).

We conducted an analysis of Longitudinal Health Insurance Database 2000 (LHID 2000), which is a random sample of one million enrollees from the NHIRD's longitudinal data spanning the period of 1997 to 2013. Diagnostic codes were collected from the International Classification of Diseases, Ninth Revision, Clinical Modification (ICD-9-CM). We extracted the top ten most common indications for SB from the primary diagnosis codes. This study was approved by the Institutional Review Board of China Medical University in central Taiwan (CMUH-104-REC2-115-R3).

### 2.2. Animals

Male Sprague Dawley rats, purchased from BioLASCO (Taipei, Taiwan), weighing 225–300 g were used in this study. A light-dark cycle of 12:12 h, relative humidity of 55% ± 5%, and room temperature of 23°C ± 1°C were maintained. Food and tap water were provided *ad libitum*. Animal use was approved by the Institutional Animal Care and Use Committee of Show Chwan Memorial Hospital (no. 106021) and followed the Guide for the Use of Laboratory Animals (National Academy Press).

### 2.3. SB Preparation

**Figure 1:**
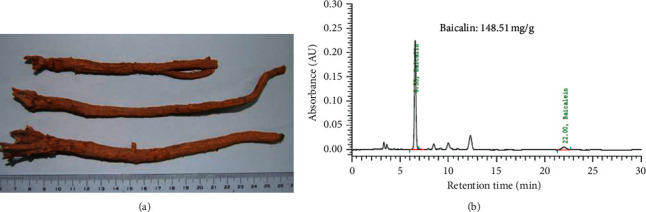
(a) Morphology of *Scutellaria baicalensis* (Huang Qin). (b) High-performance liquid chromatography (HPLC) fingerprint of *Scutellaria baicalensis* extract subtle granules.

SB extract subtle granules were produced by Ko Da Pharmaceutical Co., Ltd. (Taoyuan, Taiwan). Scutellariae radix, the dried root of *Scutellaria baicalensis* Georgi (Fam. Labiatae), was purchased from Gansu province, People's Republic of China (Figure 1(a)). The origin and voucher specimens were identified and kept by Ko Da Pharmaceutical Co., Ltd. In brief, 210 kg of Scutellariae radix was extracted in 10-fold (w/v) boiled water for 1 h followed by filtration through a 40-mesh sieve. The filtrates were collected and subjected to vacuum concentration to produce 70 kg of extracts. The excipients, including 40.6 kg of starch, 28 kg of Scutellariae radix powder, and 1.4 kg of sodium carboxymethyl cellulose, were dried in a granulator, and then the extracts were added followed by granulation. The ratio of extracts and starch in SB extract subtle granules was 1 : 1 (w/w).

Analytical high-performance liquid chromatography was performed using a Hitachi D-7000 interface equipped with an L-7100 pump, L-7455 detector, and L-7200 autosampler (Tokyo, Japan) to examine the baicalin content in the SB extract subtle granules. The test solution was prepared by mixing approximately 0.5 g of SB extract subtle granules with 30 mL mixture of acetonitrile and diluted phosphoric acid in a ratio of 28 and 72 (v/v) under heating reflux for 30 min. After centrifugation at 4000 rpm for 10 min, the supernatant was collected and added to a mixture of acetonitrile and diluted phosphoric acid at a ratio of 28 to 72 (v/v) to make up a final volume of 100 mL and then passed through a 0.45 *μ*m filter. Around 5 mg of baicalin, with purity higher than 98% as claimed by the supplier (ChemFaces, Hubei, China), was mixed with 10 mL to obtain a stock standard solution, and 2 mL of stock standard solution was added to the mixture of acetonitrile and diluted phosphoric acid at a ratio of 28 to 72 (v/v) for a final volume of 20 mL to obtain the working standard solution. Chromatographic separation was carried out on a Mightysil RP-18 column (250 × 4.6 mm, 5 *μ*m) using an isocratic solvent system comprising a mixture of acetonitrile and diluted phosphoric acid at a ratio of 28 to 72 (v/v). The ultraviolet wavelength, flow rate, injection volume, and stop time were set at 270 nm, 1.0 mL/min, 10 *μ*L, and 30 min, respectively. The content of baicalin in the SB extract subtle granules was 148.51 mg/g (Figure 1(b)).

### 2.4. Grouping

To induce migraine attacks, the rats were given an intraperitoneal (i.p.) injection of NTG (10 mg/kg) [14]. A total of 24 rats were randomly allocated into four groups (*n* = 6) as follows:

1. Control group: i.p. injection of normal saline only
2. NTG group: i.p. injection of NTG only
3. Placebo group: oral administration of 1.0 g/kg starch 30 min before i.p. injection of NTG
4. SB-1.0 group: oral administration of 1.0 g/kg SB 30 min before i.p. injection of NTG

The dose of SB used in the present study was calculated based on the report by Nair and Jacob [18]. They reported that rat equivalent dose (mg/g) = human dose (mg/g) × 6.2. We considered that an average human weighs 60 kg. Therefore, based on the clinical human dosage of 9.6 g, which is the rational dose commonly used in clinical TCM practice and suggested by pharmaceutical companies in Taiwan, we calculated the appropriate SB dosage for rats to be 1.0 g/kg.

**Figure 2:**
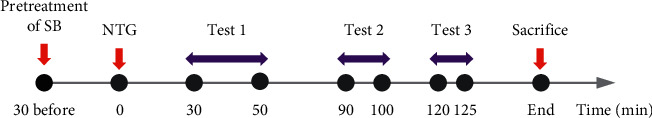
Experimental procedure. Pretreatment with *Scutellaria baicalensis* (SB): oral administration of *Scutellaria baicalensis* (SB) 30 min prior to i.p. injection with NTG; NTG: nitroglycerin (10 mg/kg) i.p. injection; test 1: rat behaviour recognition module test video recordings; test 2: light/dark box test video recordings; test 3: rough/smooth surface apparatus test video recordings.

The experimental procedure is shown in Figure 2.

### 2.5. Rat Behaviour Tests

#### 2.5.1. Assessment of Spontaneous Nociceptive Behaviour

Thirty minutes after i.p. injection with NTG, spontaneous nociceptive behaviour was observed using a transparent acrylic apparatus (45 × 45 × 35 cm^3^). A camera was placed 1 m in front of the apparatus, and the behaviour of the rats was video-recorded for 20 min. The rats' behaviour was analysed automatically using the rat behaviour recognition module of EthoVision XT 12.0 software (Noldus Information Technology, Leesburg, VA, United States).

Rat behaviour information and analysis were referenced from the descriptions in previous studies [19, 20]. In brief, rat behaviour was categorized into five types: exploratory behaviour (including rearing up and sniffing), locomotor behaviour (including walking and jumping), freezing behaviour (including twitching), resting behaviour, and grooming behaviour.

For the aforementioned behaviours, the frequency of engaging in the behaviour was calculated.

#### 2.5.2. Assessment of Light-Aversive Behaviour

Ninety minutes after i.p. injection of NTG, light-aversive behaviour was tested using a light/dark box. The light/dark box was made of two identical compartments (30 × 30 × 22.5 cm^3^), where the light chamber was placed under a bright environment without a cover, and the dark box was fully black with a lid. A small opening gate (10 cm × 10 cm^3^) connected the two chambers, and the rats could freely move across the two chambers. At the start of the assessment, the rats were placed in the centre of the light chamber and allowed to freely move across the two chambers for 10 min. The test was video-taped and analysed using EthoVision XT 12.0. The parameter of total time spent in the light chamber was further analysed.

#### 2.5.3. Assessment of Spontaneous Tactile Allodynia

One hundred and twenty minutes after the i.p. injection of NTG, spontaneous tactile allodynia was tested using a rough/smooth surface apparatus, which was modified from a previous research [21]. The apparatus consisted of a transparent acrylic box (45 × 45 × 35 cm^3^), in which the floor was divided into two identical arenas (22.5 × 45 cm^3^ each). The left-side floor surface was covered by smooth sandpaper (P1000 grit), and the right-side floor surface was covered by rough sandpaper (P40 grit). At the beginning of the test, the rats were placed in the centre of the apparatus and allowed to freely move across both arenas for 5 min. The test was video-taped and analysed using EthoVision XT 12.0. The total time spent in the smooth surface arena and heat map plots of the mean locations of the groups were calculated.

All rats were sacrificed after the completion of all behavioural tests.

### 2.6. Statistical Analysis

All the data are shown as mean ± SEM. Statistical significance between the control, NTG, placebo, and SB-1.0 groups was analysed using one-way ANOVA followed by a Tukey's post hoc test. A *p* value <0.05 was considered statistically significant. GraphPad Prism 7.0 software (San Diego, CA, USA) was used for the statistical analysis.

## 3. Results

### 3.1. Clinical Applications of SB in Taiwan

To investigate the clinical application of SB by TCM doctors in Taiwan, we conducted a population-based analysis using LHID 2000, which comprises a random sample of one million participants from the NHIRD between 1997 and 2013. The top 10 most frequent clinical applications of SB between 1997 and 2013 in Taiwan are shown in Table 1. The use of SB to treat headache (*N* = 4849, 2.51%) was the fourth most common clinical application.

### 3.2. Effect of Pretreatment with SB on NTG-Induced Spontaneous Nociceptive Behaviour in Rats

#### 3.2.1. Exploratory Behaviour including Rearing Up and Sniffing

**Figure 3:**
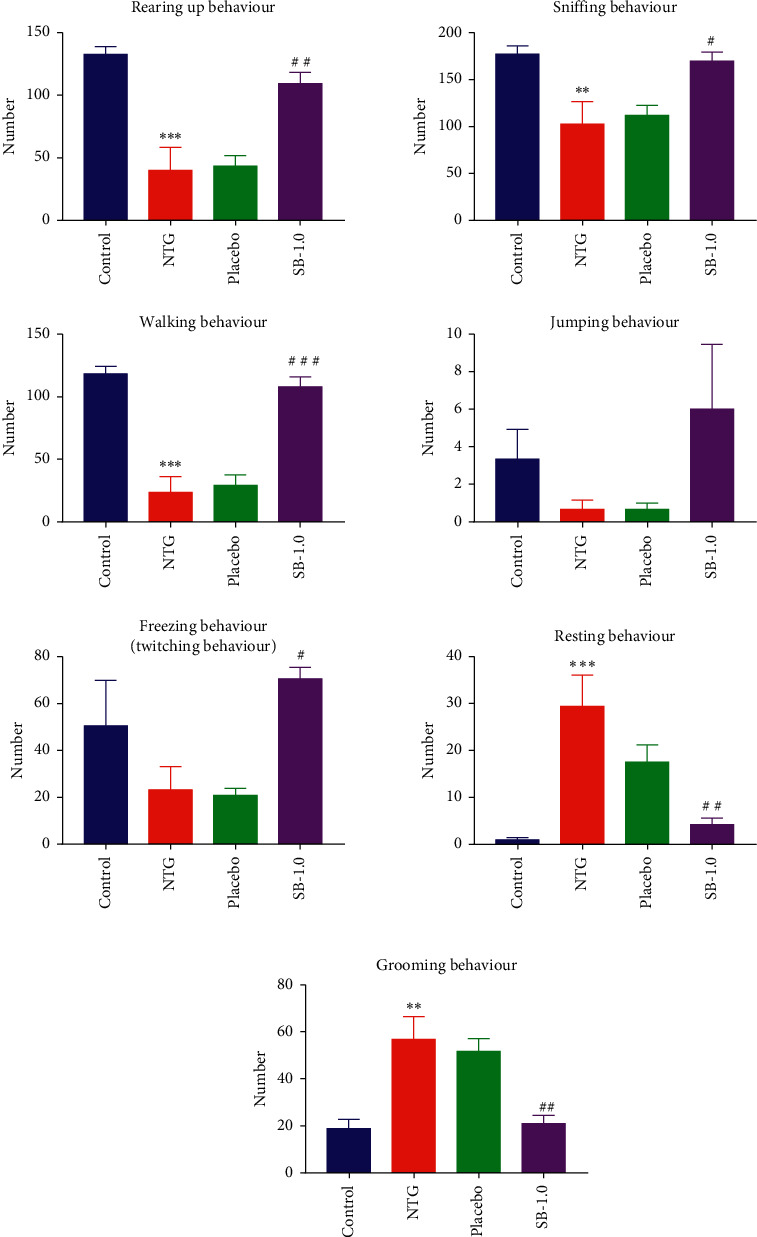
Effects of pretreatment with *Scutellaria baicalensis* (SB) on spontaneous nociceptive behaviour in a nitroglycerin-induced migraine rat model: (a) (A) the frequency of rearing up behaviour (exploratory behaviour); (B) the frequency of sniffing behaviour (exploratory behaviour). (b) (A) The frequency of walking behaviour (locomotor behaviour); (B) the frequency of jumping behaviour (locomotor behaviour). (c) The frequency of twitching behaviour (freezing behaviour). (d) The frequency of having an immobile posture or sleeping (resting behaviour). (e) The frequency of grooming the face or body (grooming behaviour). Frequency: the numbers/20 min; control: control group; NTG: NTG group; placebo: placebo group; SB-1.0: SB-1.0 group; data are presented as mean ± SEM. *∗p* < 0.05,^*∗∗*^*p* < 0.01,  and ^*∗∗∗*^*p* < 0.001 for the NTG group versus the control group; #*p* < 0.05,^##^*p* < 0.01,  and ^###^*p* < 0.001 versus the NTG group.

Notably, the rats in the NTG group engaged less in rearing up behaviour than those in the control group did (*p* < 0.001; Figure 3(a) (A)). Pretreatment with 1.0 g/kg SB (*p* < 0.01; Figure 3(a) (A)), but not placebo (*p* > 0.05; Figure 3(a) (A)), resulted in relatively more frequent rearing up behaviour compared to that in the NTG group.

Similar to the above outcome, the rats in the NTG group did not engage in sniffing behaviour as frequently as those in the control group did (*p* < 0.01; Figure 3(a) (B)). Pretreatment with 1.0 g/kg SB (*p* < 0.05; Figure 3(a) (B)), but not placebo (*p* > 0.05; Figure 3(a) (B)), resulted in a more frequent sniffing behaviour compared to that of the NTG group.

#### 3.2.2. Locomotor Behaviour including Walking and Jumping

The rats in the NTG group engaged in walking behaviour less frequently than the control group (*p* < 0.001; Figure 3(b) (A)). Pretreatment with 1.0 g/kg SB (*p* < 0.001; Figure 3(b) (A)), but not with placebo (*p* > 0.05; Figure 3(b) (A)), resulted in a higher frequency of walking behaviour in comparison with that of the NTG group.

No significant differences were noted between the control, NTG, placebo, and SB-1.0 g/kg groups (all *p* > 0.05; Figure 3(b) (B)) in the analysis of the frequency of jumping behaviour.

#### 3.2.3. Freezing Behaviour including Twitching

The rats in the NTG group did not show significantly different twitching behaviour from that of rats in the control group (*p* > 0.05; Figure 3(c)). Pretreatment with 1.0 g/kg SB led to more frequent twitching behaviour compared to that in the NTG group (*p* < 0.05; Figure 3(c)). However, pretreatment with placebo resulted in a similar frequency of twitching behaviour as that in the NTG group (*p* > 0.05; Figure 3(c)).

#### 3.2.4. Resting Behaviour

The prevalence of resting behaviour was higher in the rats in the NTG group than that in the control group (*p* < 0.001, Figure 3(d)). Pretreatment with 1.0 g/kg SB (*p* < 0.01; Figure 3(d)), but not placebo (*p* > 0.05; Figure 3(d)), resulted in lower engagement in resting behaviour compared to that in the NTG group.

#### 3.2.5. Grooming Behaviour

The rats in the NTG group exhibited grooming behaviour more frequently than the control group did, and the difference was significant (*p* < 0.01; Figure 3(e)). Pretreatment with 1.0 g/kg SB (*p* < 0.01; Figure 3(e)), but not with placebo (*p* > 0.05; Figure 3(e)), resulted in a decrease in the frequency of this behaviour.

#### 3.2.6. Effect of Pretreatment with SB on NTG-Induced Light Aversion in Rats

**Figure 4:**
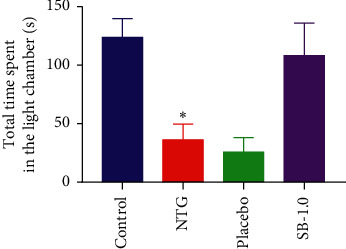
Effects of pretreatment with *Scutellaria baicalensis* (SB) on total time spent in the light chamber (s/10 min) of light-aversive behaviour in a nitroglycerin-induced migraine rat model. Control: control group; NTG: NTG group; placebo: placebo group; SB-1.0: SB-1.0 group; data are presented as mean ± SEM. *∗p* < 0.05, *∗∗p* < 0.01, and *∗∗∗p* < 0.001 for the NTG group versus the control group; #*p* < 0.05,^##^*p* < 0.01,  and ^###^*p* < 0.001 versus the NTG group.

The rats in the NTG group spent significantly less time in the light chamber compared to that spent by the control group (*p* < 0.05; Figure 4). Rats pretreated with 1.0 g/kg SB showed a difference in behaviour that approached marginal significance (*p*=0.054; Figure 4), which was not observed with placebo pretreatment (*p*=0.977; Figure 4), and spent a longer time in the light chamber than rats in the NTG group did.

#### 3.2.7. Effect of Pretreatment with SB on NTG-Induced Spontaneous Tactile Allodynia Behaviour in Rats

**Figure 5:**
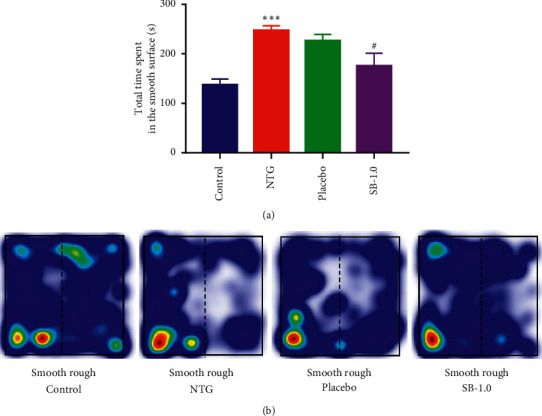
Effects of pretreatment with *Scutellaria baicalensis* (SB) on spontaneous tactile allodynia behaviour in an NTG-induced migraine rat model. (a) Total time spent engaging in spontaneous tactile allodynia behaviour on the smooth surface (s/5 min); (b) group mean heat map plot (the left side is the smooth surface arena). Control: control group; NTG: NTG group; placebo: placebo group; SB-1.0: SB-1.0 group; smooth: smooth surface; rough: rough surface. Data are presented as mean ± SEM. *∗p* < 0.05, *∗∗p* < 0.01, and *∗∗∗p* < 0.001 for the NTG group versus the control group; #*p* < 0.05,^##^*p* < 0.01,  and ^###^*p* < 0.001 versus the NTG group.

The rats in the NTG group spent considerably longer time on the smooth surface than those in the control group did (*p* < 0.001; Figure 5(a)). The time spent engaging in this behaviour was decreased by pretreatment with 1.0 g/kg SB (*p* < 0.05; Figure 5(a)). Group mean location heat maps demonstrated that rats in the NTG group had a higher preference for the arena border and corners and spent more time on the smooth surfaces than those in the control group did (Figure 5(b)). This behaviour was decreased by pretreatment with 1.0 g/kg SB (Figure 5(b)).

## 4. Discussion

Migraine is considered a headache disorder, which involves the neural and vascular components of nociceptive transmission, and is associated with multiple pathophysiology, such as inflammation, and impaired functioning of neurotransmitters, ion channels, immune system, mitochondrial function, and oxidative stress factors [22]. Although Western medicine has rapidly developed treatments for migraine, some patients who face adverse effects of these medications seek alternative therapies for migraine prophylaxis and treatment [23]. Based on TCM theories and records in Chinese historical book, SB is considered an excellent anti-inflammatory and analgesic drug in the experience of traditional Chinese doctors; its use can be traced to the book “*Shen Nong Ben Cao Jing*” written in the Han Dynasty [8]. Moreover, SB is not only widely used in China but is also used as a medicinal plant in many countries around the world. For example, SB is a traditional plant medicine applied to wounds and insect bites in Nepal [8]. The chemical components, such as flavonoids, diterpenes, polyphenols, and amino acids, of SB [24] have several important biological activities, including anti-inflammatory, antitumor, antioxidant, antibacterial, and antiviral effects [25]. In addition, a recent study showed that SB has potential therapeutic effects on coronavirus disease 2019 [26]. Moreover, baicalin and baicalein, which are the major bioactive compounds in SB, have demonstrated an important role in inhibiting the production of inflammatory cytokines [25, 27], NF-*κ*B signalling [28, 29], and c-fos expression [30] in multiple experimental models. These mechanisms are crucial in the pathophysiological processes of migraine. Hence, we assumed that SB could be potentially effective in migraine treatment.

However, to date, there has been no large-scale exploration of the actual clinical use of SB, including the distribution and frequency of use. In Taiwan, Chinese medicine is a popular medicine system for people. We also have a robust healthcare system with complete database records. Hence, we conducted a retrospective nationwide investigation of the prescription practices of TCM doctors to understand the actual clinical application of SB. Our findings revealed that SB was recommended for a variety of diseases, mainly respiratory inflammatory disorders, headache, sleep impairments, and gastrointestinal discomfort, in Taiwan. These findings are similar to those of a previous study, which showed that SB is commonly used for and has excellent therapeutic effects in sleep disturbance, hepatitis, diarrhoea, vomiting, haemorrhage, hypertension, and respiratory infection [8]. We found that SB could be used in varied diseases and is not limited to specific conditions; hence, the percentage of top ten diseases where SB was used was not particularly high. Although only 2.51% of the indications (the fourth most common indication) of SB in Taiwan was for the treatment of headaches, including migraine, surprisingly, it was not included in the clinical or basic applications of SB in previous scientific reports. It should be noted that our clinical investigation of the indications for SB usage is very credible because we used a nationwide random sample from the NHIRD, which had minimal selection bias owing to the very high rate of insured individuals and the fact that all Chinese herbal products were prescribed by well-trained and qualified TCM practitioners in Taiwan. Hence, we further designed an animal experiment using the widely accepted NTG-induced migraine model to prove the effectiveness of SB.

In the present study, we assessed various migraine-like behaviours of NTG-induced migraine rat models by using recorded videos that were analysed by reliable automated software [31, 32]; therefore, the results of the present study could be considered more credible and more objective than manually scored behaviours.

Migraine diagnosis is based on clinical self-reported pain and attendant symptoms. Although animals are not verbal, recent studies have considered NTG-induced animal models as clinically relevant due to the translational ability of behavioural endpoints [12, 21]. Our previous studies showed that NTG-induced nociceptive behaviours in rats with migraine headache included reduced exploratory and locomotor behaviours and increased resting behaviour [33, 34]. The aforementioned nociceptive behaviours in rodents mimicked those experienced by people with migraine, who generally present with reduced involvement in routine physical activity and decreased interest in exploring new surroundings or objects during an acute migraine attack episode [34, 35]. Therefore, we could use the parameters of the rat model to test antianalgesic drug potency. The results of the present study demonstrated that pretreatment with 1.0 g/kg SB could increase locomotor activity and decrease resting behaviour in NTG-induced migraine rats, suggesting that pretreatment with SB could alleviate migraine pain in humans.

Photophobia is one of the important symptoms of migraine [36]. Previous reports have shown that light aversion behaviour, tested using the light/dark box, in NTG-induced migraine rodents effectively mimics the phenomenon of photophobia [35, 37]. Our results indicated that pretreatment with 1.0 g/kg SB resulted in decreased light aversion, which approached marginal significance, implying that SB might alleviate photophobia in an individual who experiences migraine.

Cutaneous allodynia is a common symptom seen in migraineurs; it indicates cranial hypersensitivity and appears to be a predictor of migraine chronification [38, 39]. Recently, a rough/smooth apparatus test was used to evaluate spontaneous tactile allodynia in an NTG-induced migraine rodent model [21]. Our results showed that pretreatment with 1.0 g/kg SB decreased the total time spent on the smooth surface in the rough/smooth test, indicating that SB can decrease tactile allodynia in NTG-induced migraine rat models, which suggests that SB can be helpful for the clinical treatment of cutaneous allodynia in migraineurs.

There are still some limitations to the present study: (1) the animal experiment lacked a group treated with a medicine, such as ibuprofen, to compare the efficacy of SB and conventional Western medicine. In addition, a dose-dependent relationship of the bioactive agents of SB, such as baicalin, was not reported in the current animal experiment; (2) the regulatory mechanisms of SB in migraine rats were not explored, and further validation, such as by biomarker determination (NO, CGRP level) and immunohistochemistry assay, should be conducted to investigate neuronal activity in the future; and (3) although the results of the present study explain how SB was helpful for the treatment of NTG-induced migraine in rats, a well-designed clinical trial is required to evaluate the efficacy of SB in humans.

## 5. Conclusions

The results of the present study indicated that pretreatment with SB increased the frequency of rearing up and sniffing in exploratory behaviour and increased the frequency of walking in locomotor behaviour in NTG-induced migraine models. NTG-induced migraine rats pretreated with SB also had lower frequencies of both resting behaviour and grooming behaviour. These phenomena indicate that SB could be helpful in decreasing the migraine pain level. Additionally, NTG-induced migraine rats pretreated with SB spent less time on the smooth surface compared to the time spent by the rats that did not receive SB pretreatment, which indicates that SB could be helpful in decreasing cutaneous allodynia in migraineurs. These results support the application of SB for the treatment of headache, as is a common practice in Taiwan.

## Figures and Tables

**Table 1:** Top 10 clinical applications of *Scutellaria baicalensis* by traditional Chinese medicine doctors from 1997 to 2013 in Taiwan.

Ranking	ICD-9-CM	Indications	Usage number (*N*)	Percentage (%)
1	460	Acute nasopharyngitis (common cold)	25828	13.37
2	786.2	Cough	15476	8.01
3	477.9	Allergic rhinitis cause unspecified	4869	2.52
4	784.0	Headache	4849	2.51
5	780.59	Other sleep disturbances	2655	1.37
6	490	Bronchitis, not specified as acute or chronic	2483	1.29
7	472.0	Chronic rhinitis	2472	1.28
8	780.50	Sleep disturbances, unspecified	2445	1.27
9	536.9	Unspecified functional disorder of stomach	2339	1.21
10	536.8	Dyspepsia and other specified disorders of function of stomach	2255	1.17

ICD-9-CM: International Classification of Diseases, Ninth Revision, Clinical Modification.

## Data Availability

The datasets used and analysed during the current study are available from the corresponding author upon reasonable request.

## Abbreviations

i.p.: Intraperitoneal injection
ICD-9-CM: International Classification of Diseases, Ninth Revision, Clinical Modification
NHIRD: National Health Insurance Research Database
NTG: Nitroglycerin
SB: *Scutellaria baicalensis*
TCM: Traditional Chinese medicine.
